# Livestock Helminths in a Changing Climate: Approaches and Restrictions to Meaningful Predictions

**DOI:** 10.3390/ani2010093

**Published:** 2012-03-06

**Authors:** Naomi J. Fox, Glenn Marion, Ross S. Davidson, Piran C. L. White, Michael R. Hutchings

**Affiliations:** 1 SAC, West Mains Road, Edinburgh, EH9 3JG, UK; E-Mails: naomi.fox@sac.ac.uk (N.J.F); ross.davidson@sac.ac.uk (R.S.D); 2 Environment Department, University of York, Heslington, York, YO10 5DD, UK; E-Mail: piran.white@york.ac.uk; 3 Biomathematics and Statistics Scotland, Kings Buildings, Edinburgh, EH9 3JZ, UK; E-Mail: glenn@bioss.ac.uk

**Keywords:** climate change, prediction, risk, livestock, parasites, helminths, disease, modelling

## Abstract

**Simple Summary:**

Parasitic helminths represent one of the most pervasive challenges to livestock, and their intensity and distribution will be influenced by climate change. There is a need for long-term predictions to identify potential risks and highlight opportunities for control. We explore the approaches to modelling future helminth risk to livestock under climate change. One of the limitations to model creation is the lack of purpose driven data collection. We also conclude that models need to include a broad view of the livestock system to generate meaningful predictions.

**Abstract:**

Climate change is a driving force for livestock parasite risk. This is especially true for helminths including the nematodes *Haemonchus contortus*, *Teladorsagia circumcincta*, *Nematodirus battus*, and the trematode *Fasciola hepatica,* since survival and development of free-living stages is chiefly affected by temperature and moisture. The paucity of long term predictions of helminth risk under climate change has driven us to explore optimal modelling approaches and identify current bottlenecks to generating meaningful predictions. We classify approaches as correlative or mechanistic, exploring their strengths and limitations. Climate is one aspect of a complex system and, at the farm level, husbandry has a dominant influence on helminth transmission. Continuing environmental change will necessitate the adoption of mitigation and adaptation strategies in husbandry. Long term predictive models need to have the architecture to incorporate these changes. Ultimately, an optimal modelling approach is likely to combine mechanistic processes and physiological thresholds with correlative bioclimatic modelling, incorporating changes in livestock husbandry and disease control. Irrespective of approach, the principal limitation to parasite predictions is the availability of active surveillance data and empirical data on physiological responses to climate variables. By combining improved empirical data and refined models with a broad view of the livestock system, robust projections of helminth risk can be developed.

## 1. Introduction

Climate change has been implicated as a driving force for recent parasite range expansions, and efforts have been made to model the relationship between pathogen levels and climate. The most economically important parasitic helminths of livestock in temperate climes include the nematodes *Haemonchus contortus*, *Teladorsagia circumcincta* and *Nematodirus battus*, and the trematode *Fasciola hepatica*. The increase in these helminths in recent years [1,2,3,4,5] has been attributed to climate change, since the survival of the free-living stages is chiefly affected by temperature and moisture, and larval development rate is highly temperature dependent [6,7,8,9].

The development of evidence-based risk assessments and targeted surveillance are pivotal when the welfare and economic costs of these pathogens are considered. Subclinical infection is characterised by weight loss, lower milk yield, loss of condition, abortion, infertility and veterinary costs, and heavy infections can cause host mortality. Indirect economic losses from pathogen outbreaks are also incurred via export restrictions and surveillance and mitigation costs [10]. Despite the deleterious impacts of helminths on the livestock industry and their dependence on climatic conditions, predictions of long-term threats to animal health from climate change have so far concentrated on heat stress [11,12,13,14,15,16] and viruses spread by volant vectors, such as blue tongue [17,18,19,20,21,22]. Although there have been a number of studies aiming to link the recent changes in helminthiasis abundance and distribution with environmental change [1,3,4,5,23,24,25], there is a lack of predictions for future helminth risk to livestock.

Here we explore the optimal approaches to generating long-term predictions for helminth risk, stratifying modelling approaches as either correlative or mechanistic. We also highlight the obstacles to generating meaningful predictions and the need for a multidisciplinary approach.

## 2. Modelling the Change

### 2.1. The Correlative Approach

Predictive models of species distribution, for both epidemiology and conservation, are often based on correlative ecological niche models [26,27,28,29,30,31,32]. These models are based on Hutchinson’s ecological niche theory where the current geographic distribution is used to infer the environmental requirements of a species [33]. The current, past or future distribution of that species is then predicted based on these requirements [34]. Insights into the biology of parasite dynamics should be used to systematically build the foundations of these models, and the most proximal environmental predictors should be chosen based on known ecological and physiological theory [35].

A number of programmes are available to determine a species’ climate envelope by matching current distribution with climatic parameters, such as CLIMEX [36], HABITAT [37], DOMAIN [38] and SPECIES [39]. In addition to these generic models, species-specific correlative models have also been developed, however these models have primarily been applied to species of conservation importance and invasive alien species. To date, correlative predictive models of helminthiasis have focussed on *F. hepatica* (liver fluke) due to the close relationship between weather and fasciolosis outbreaks [2] and the worldwide importance of fasciolosis as a zoonosis. The UK’s National Animal Disease Information Service (NADIS) and the Department of Agriculture and Food in Ireland currently predict *F. hepatica* incidence using the Ollerenshaw index, which is a correlative model focusing on temperature and moisture availability [40,41]. Through applying a modified Ollerenshaw index to UKCP09 climate projections, the first long-term predictions of future *F. hepatica* risk for 2020–2070 across the UK have been developed [42]. The resultant risk maps predict unprecedented levels of fasciolosis outbreaks, and possible changes in the timing of disease outbreaks due to increased risk from overwintering larvae. Despite an overall long term increase in all regions of the UK, spatio-temporal variation in risk levels is expected, with infection risk due to reduce in some areas and fluctuate greatly in others. This forecast is the first approximation of the potential impacts of climate change on helminth risk in the UK, and indicates where active disease surveillance should be targeted.

There are a number of factors that made correlative modelling the preferred approach for determining fascioliasis risk. Firstly, the existence of long term prevalence data facilitated the development of a statistical model [41]. Secondly, despite their complex life cycle, the distribution of liver fluke is driven by simple proximal drivers—temperature and water availability. It is these drivers that form the basis of the correlative model. Thirdly, their dispersal by wild hosts and speed of colonisation of new regions makes them ubiquitous where livestock are present across their fundamental niche.

Despite the information that correlative models can provide, there are a number of disadvantages to applying this approach to climate change predictions. A prominent bottleneck to the development of correlative models is the lack of current or past parasite distribution data. The data used to train these models is usually opportunistic, passive surveillance data. Climate parameters are used according to which measurements and predictions are available, rather than those that are most pertinent to disease transmission. In order to build reliable models, purpose-driven active surveillance data is needed. As climate affects long term, large scale trends, these data must be collected over appropriate scales to observe these trends, rather than the patchy distribution visible at finer scales, which is likely to be a consequence of non-climatic factors. It is equally important to apply predictive models at the right scale. It is often tempting to apply models on as fine a scale as the climate projections allow, as this will lead to detailed maps with an illusion of greater accuracy. However this temptation should be resisted and models based on climatic parameters alone should only be applied at an appropriately coarse scale.

The lack of distribution data also impedes the validation of these predictive models, as ideally they should be validated with data fully independent from that used to build the model [26,28,43]. A further issue with validation is that it often indicates the models ability to predict current distribution; models that are effective at predicting current distribution may not be so reliable when predicting future outbreaks [34]. Hence there is a need to validate these models with data outside the spatial and temporal range of the training dataset. To make use of existing datasets for both model creation and validation there are ongoing developments in statistical methodology to account for biases in reported data. Existing datasets can be utilised for modelling distributions if imperfect detection is considered, with irregular sampling intensity and false absences accounted for using hierarchical Bayesian models [29,30,44,45]. Uncertainty in predictor variables can also be addressed through Bayesian analysis [46].

Given sufficient data for creation and validation of correlative models, fundamental disadvantages remain. As correlative models are generated using distribution data they are based on the realized niche [47] which includes competitive exclusion and biotic interactions (e.g., the presence of hosts and other pathogens), rather than the whole fundamental niche. As distribution data are usually only available for a limited area or time window, correlative models are generally fitted using data reflecting a snapshot of the current climate-pathogen relationship. An assumption is therefore made that a species has reached equilibrium with its environment [35]. This ignores any non-climatic dispersal constraints that may be restricting the current range, or declining sink populations where presence is recorded but climate is not actually suitable for long-term population survival. This failure to account for non-equilibrium ranges is an inherent weakness in correlative models [48,49]. This weakness could be especially pertinent for emerging parasites which are unlikely to be in equilibrium with their current environment, so could be absent from climatically suitable areas due to low propagule pressure. Conversely, these models will not identify areas which are not within bounds necessary for pathogen survival, but where deleterious levels of parasitism could be reached given sufficient propagule pressure. This issue posed by the non-equilibrium nature of emerging pathogens could be addressed through training models for emerging parasites using distribution data from their long established native range [35].

Above all aforementioned limitations, the dangers in extrapolating statistical models are especially pertinent to climate projections. The assumption that a species is in equilibrium with its current environment and the reliance on relationships between climate variables that may not exist in novel climate change scenarios leads to equivocal results when models are extrapolated spatially and temporally [29,44,50,51]. We are likely to experience previously unseen climate combinations and climate parameters exceeding their current observed ranges; parasites could be well adapted to these novel situations, however correlative models would assume these conditions to be unsuitable [34]. Additionally, correlative models cannot identify points where the system behaviour undergoes a qualitative change (tipping points). It is this inability to extrapolate that further emphasises the need for an alternative modelling approach.

As a further testament to correlative models’ often unreliable outputs, models created using different statistical approaches have been shown to produce conflicting distribution estimates, even when trained with the same distribution and climate data [52,53]. Such differences between distribution estimates can be exacerbated when extrapolating to novel climatic conditions [32]. To address the divergence in model outputs, a framework for selecting the most robust statistical modelling technique has been developed [32]. This approach would be useful for selecting optimal models for predicting changes in helminth distribution. The differences between distributions predicted by different models could also be informative; discovering why predictions differ could improve understanding of the main drivers, and quantification of the differences could inform decision making and risk analysis [29].

Despite their limitations, correlative models can still provide a first indication of how climate will influence helminth distribution, and identify where limited resources and targeted surveillance should be focused. They provide a useful tool when too few elements of the transmission process have been quantified for the creation and parameterisation of mechanistic models. The limitations of correlative models are being addressed through continued development of statistical methodologies, led by work in both conservation and invasive species control; there is scope for these emerging approaches to be applied to predicting livestock parasite risk. Reliability of correlative models will ultimately be governed by the quality of data used for model training and validation, the statistical methods employed, the ecological and physiological knowledge on which inclusion of proximal variables is based, and discrepancies between the realized and fundamental niche.

### 2.2. The Mechanistic Approach

The second approach to understanding and predicting parasite risk is the process-based mechanistic approach. Mechanistic models are based on detailed knowledge of the physiology of the species [34] and attempt to replicate the underlying mechanisms that drive the species’ response to environmental variables [54]. Given sufficient understanding of the parasites physiology, these models can be employed to predict changes in prevalence [55]. Previous mechanistic models have explored the dynamics of helminth infection in livestock [56,57,58,59,60,61,62,63,64,65]. However these models have not been used to assess the impacts of climate change on helminth transmission.

One element of mechanistic modelling that makes it well adapted in a changing climate is that it is less prone to extrapolation problems than correlative models. The mechanistic approach does not rely on relationships between climate variables that may cease to exist under future climate change [34,47,66,67], making them less prone to breaking down when tested outside current observation limits. Consequently, the mechanistic approach is considered superior in extrapolating beyond current conditions and forecasting the impacts of climate change [68]. When predicting a parasite’s response to novel conditions it is vital that models account for the possibility of non-linear responses to predictor variables [43]. Through developing a mechanistic model looking at the effects of increasing temperatures on Schistosomiasis prevalence and abundance, Mangal *et al.* (2008) found that *Schistosoma mansoni* showed a non-linear response to temperature change [68]. Extrapolated predictions for novel climates may prove to be unreliable if the response curves are not fully determined, hence empirical data on survival and development under conditions beyond a parasite’s current climatic ranges are required.

The mechanistic approach also avoids some of the inherent problems that correlative models have when applied to emerging and invasive parasites since mechanistic models do not assume the species is in equilibrium with the environment [48] and are capable of predicting tipping points. By incorporating key mechanisms into the model structure more complex questions can also be addressed, such as determining the impact of climate on specific stages of the lifecycle.

The severity of helminth infections is often dependent on intensity, rather than prevalence. The economic and welfare implications of outbreaks would be difficult to evaluate using a correlative approach, as data availability typically constrains these models to only look at prevalence. If data on adult worm burden are available they are often based on indirect measures such as faecal egg count. It is more feasible to include models of adult worm burden within a mechanistic framework. Although mechanistic models can predict changing intensity, the relationship between helminth burden and host production losses is non-linear; a doubling of infection intensity does not lead to a doubling of production loss [69]. A deeper understanding of the relationship between parasite burdens and potential production losses is still required.

The survival of larvae on pasture is largely driven by climate [62], but helminth population dynamics are governed by density dependent process and it is vital that these processes are incorporated (e.g., host acquired immunity), as more larvae on pasture do not necessarily translate to higher parasite burden in the livestock [64]. Previous parasite models have concentrated on infection dynamics in a single host, returning the average host infection level and assuming each host interacts with its own private population of parasites [57,59,62,64]. In reality, aggregation of parasites in host populations is a ubiquitous phenomenon [70]. It has been proposed that heterogeneities in host infection levels arise due to numerous factors including variations in host resistance and susceptibility due to behavioural or physiological differences and spatial heterogeneity in infection risk [71,72,73,74]. Average host models ignore this overdispersion of helminths; a potentially critical feature in a system heavily driven by density-dependent processes.

By modelling the average host, it is assumed that the same level of regulatory constraint is experienced by all parasites in the population. In an overdispersed population, aggregation results in an increase in the effective density experienced by individual parasites, increasing the influence of regulatory processes [74,75,76]. These regulatory processes drive a parasite population towards equilibrium [63], and hence aggregated populations reach equilibrium more quickly and at a lower level. As a consequence, models that do not take the overdispersed parasite distribution into account are likely to overestimate burden [75,76]. Variation in host worm burden within a herd has been generated artificially in infection models through assuming a fixed distribution of worms across hosts, and the system having a pre-defined degree of overdispersion [56,75]. There is scope to develop models where an overdispersed distribution is an emergent property, and which subsume both the mechanisms that generate aggregation and the impacts of the resultant aggregation on infection dynamics. Future changes in grazing season, stocking density, host behaviour and host resistance may affect the aggregation of parasites at the suprapopulation level (defined as all parasites of a given species, at all stages of development, within an ecosystem [77]). Models that incorporate the changing distribution of the suprapopulation would also allow identification of scenarios where individuals within a herd are at risk from heavy infections, thus addressing economic and welfare concerns.

Unlike correlative models, mechanistic model development is not restricted by the paucity of accurate distribution data. Instead, the primary restriction to their development is the extensive physiological data needed for parameterisation [35]. Recent empirical work on how livestock parasites respond to changing climatic variables should facilitate their development and parameterisation. For example, the correlation between the timing, distribution and quantity of rainfall and *H. contortus* development have been investigated [6] and threshold rates of precipitation and evaporation required for survival of *H. contortus* larvae to the infective stage have been determined [78]. The impact of temperature on the emergence of the infective stages of trematodes from the intermediate snail hosts have also been reviewed [79,80]. These studies demonstrate that there are quantitative data on the effects of climatic variables on physiological processes, which can be used to parameterise mechanistic models.

In addition to altered temperature and precipitation, changes in UV reaching the extra-mammalian stages will also vary with changing climate (due to e.g., varying cloud cover, changes in development window and seasonality of infection). Van Dijk *et al.* (2009) investigated how ultraviolet (UV) light affects Trichostrongyloid nematodes, *H. contortus*, *N. battus* and *T. circumcinta*, in their infective L3 stages [81]. Mortality rates increased by up to 100 times due to exposure to UVA light equivalent to the maximum levels expected for a summers day. There were inter-species differences observed: *N. battus*, with its arctic origins, and *T. circumcincta* were less resistant than the tropic-adapted *H. contortus* [81]. Previous mechanistic models have focused on changing temperature, as this was the parameter most studied and available in climate projections. However this study highlights the importance of including other environmental variables in predictive models.

Another oversight in some mechanistic models is that they assume Liebig’s Law of the Minimum [34], which presumes that the overall response will be determined by the most limiting factor. This is not necessarily true. For example, larvae on pasture could survive otherwise deleteriously low levels of rainfall if temperatures were not too high. This emphasises the need to look at proximal, rather than distal variables—in this case water availability rather than levels of rainfall. It also emphasises the importance of looking at variables in combination.

In contrast with the correlative approach, mechanistic models are based on measured physiological and behavioural parameters, and reflect the fundamental rather than realized niche [35]. This leads to over estimation of risk showing the whole potential range if dispersal restrictions or biotic interactions are not accounted for [34,48,82,83]. The extent of predictive errors will be partly dependent on the proportion of the fundamental niche that the realized niche occupies [35]. This proportion will depend on the parasites dispersal and competitive abilities; if the discrepancies between the species’ fundamental and realized niche could be identified, it could give an indication of how accurate predictive models could be for particular species.

To attain realistic predictions of species distribution, models should ultimately integrate constraints from biotic interactions and dispersal [35]. Projections often assume either complete dispersal, where the parasite can reach all areas with permissive climes, or no dispersal where presence is only predicted in areas of its current range where climate remains within viable thresholds [35]. It is overly simplistic to assume that a species will either fully disperse to fill the fundamental niche, or that they will only survive in areas where the projected fundamental niche and current realized niche overlap [26], however, this approach does at least provide (often very wide) bounds on the possible distribution. The importance of incorporating species interactions in prediction models has been demonstrated [51,84,85], with biotic interactions influencing the predictive power of correlative models even at the macro-ecological scale [85]. The incorporation of dispersal also influences predictive power [30,31,86], with the disparity between models that do incorporate dispersal and those that do not increasing under more extreme climate warming scenarios [31]. The integration between model projections, biotic interactions and simulated host/pathogen distribution and dispersal patterns would improve the accuracy of predictive models (both correlative and mechanistic). This could be achieved through the adoption of hierarchical Bayesian frameworks [86]. However, these should take into account that biotic interactions and dispersal abilities could change with time. Due to the high selective pressures that novel conditions are likely to impose, climate change is likely to select for phenotypes with enhanced dispersal capabilities that can track the shift in their climate envelope. This would affect the parameterisation of models that incorporate dispersal.

Mechanistic models provide a powerful tool in predicting the influence of climate change on helminth risk. They are comparatively robust under spatio-temporal extrapolation and can be developed to answer complex questions. However, a move towards a mechanistic approach should not be seen as a way of alleviating the need for distribution data; to develop and validate models and assess their continued reliability under changing conditions, there is a need for ongoing surveillance.

## 3. Looking Beyond Climate: The Need for a Panoptic View

Climate is an important driver of helminth distribution, however transmission to livestock at the farm level is also controlled by husbandry. Livestock husbandry is ever changing and the adoption of mitigation and adaptation strategies will be necessary under continuing environmental change. Methods for alleviating heat stress in cattle have been explored including shade provision [14] and different feeding and sprinkling regimes [87]. Changes in the time of highest feed production may alter, resulting in timing of livestock reproduction being managed differently [88]. Housing outdoors instead of indoors in winter will also affect parasite transmission cycles [16]. Additionally, as certain breeds are more susceptible to extreme weather conditions, the breed structure of livestock may have to change to ensure that production levels are maintained and animal welfare needs met.

The influence of these adaptation and mitigation strategies on parasite risk has yet to be incorporated in pathogen transmission models. There is a propensity for predictive models to incorporate ever refined climate prediction, however these will not provide meaningful indications of future risk if they are parameterised for outdated management approaches and redundant host population structures. Predictive models need to have the architecture to allow adaptation and mitigation strategies to be incorporated. For example, as different breeds have varying levels of disease susceptibility [10,13,89], the differing levels of resistance for future breed structures need to be considered in transmission models.

The architecture of mechanistic models should also allow more complex issues to be addressed, for example, determining which of the panoply of potential control strategies resources should be invested into developing. There is currently a strong reliance on anthelmintics for controlling helminths in intensively grazed livestock [90], either through targeting the number of adult parasites in the host, or the number of infectious larvae on pasture. However development of anthelmintic resistance is an ongoing problem [5,23,91,92,93,94] and resistance development could be accelerated in a warming climate [90]. As anthelmintics become decreasingly efficient in combating these parasites, strategies to lower the dependence on chemicals and manage the spread of resistance need adopting [95,96,97,98,99]. There are a variety of alternative control strategies, often linked with organic production. These include grazing management strategies to decrease the number of infective larvae that the host is exposed to, and breeding for resistance in the host [100,101,102,103]. Models should be developed with the architecture to consider interacting effects of changes in climate, management and control strategies.

## 4. Conclusions and Recommendations

Although correlative modelling is a useful tool for establishing baseline predictions, a drive towards mechanistic process-based models will ultimately be needed if we are to foresee the consequences of subtle interactions between various components of a system under climate influence. Although laboratory studies have been done to aid the creation of mechanistic models, the findings have not yet been utilised. To improve the reliability of extrapolating and applying models to novel climates, empirical evidence is also needed on how helminths respond when exposed to conditions beyond their current observed ranges, to account for any non-linearity.

Modelling approaches can be broadly stratified as correlative or mechanistic, but in practice it is difficult to draw an absolute distinction between these modelling techniques. Ultimately, an integration of both modelling approaches may be required. Future improvements in predictions should arise from the continued development of a hybrid approach that combines both mechanistic processes and physiological thresholds with correlative bioclimatic modelling. [28,50,51,104]. Irrespective of the modelling approach, the quality of predictions is critically dependent on the quality of available data [105]. The continued collection of active surveillance data and empirical data on physiological responses to climate variables will ultimately drive the development and validation of meaningful predictions.

The recent expansion of helminth ranges has been mirrored by our increased understanding of the role of climate in their transmission dynamics. However, climate is just one factor affecting disease ecology. The application of mitigation and adaptation strategies, combined with changing control options, need to be considered when determining the overall impacts of climate change. By combining improved empirical data and refined models with a broader view of the livestock system, projections of future disease threats can be improved. This will increase our understanding of these complex systems, identifying potential risks and highlighting opportunities for control.
